# Emphysematous pyelonephritis in an infant from Sokoto, north-western Nigeria

**DOI:** 10.4102/ajlm.v10i1.1181

**Published:** 2021-04-26

**Authors:** Fatima B. Jiya, Paul K. Ibitoye, Nma M. Jiya, Maryam Amodu-Sanni, Yahaya Mohammed, Dada M. Aquib, Lukman K. Coker

**Affiliations:** 1 Department of Paediatrics, Usmanu Danfodiyo University Teaching Hospital, Sokoto, Nigeria; 2 Department of Medical Microbiology and Parasitology, Usmanu Danfodiyo University Teaching Hospital, Sokoto, Nigeria; 3 Department of Radiology, Usmanu Danfodiyo University Teaching Hospital, Sokoto, Nigeria

**Keywords:** Emphysematous pyelonephritis, infection, kidney, infant, Sokoto

## Abstract

**Introduction:**

Emphysematous pyelonephritis is a life-threatening necrotising bacterial infection of the kidneys. It is rare among children and can be fatal if not promptly identified and treated.

**Case presentation:**

A 7-month-old male infant presented to the Emergency Paediatric Unit of Usmanu Danfodiyo University Teaching Hospital, Sokoto, Nigeria, on 12 November 2019 with a 5-day history of fever and vomiting, and a 3-day history of a progressively enlarging, left-side abdominal mass. There was associated excessive crying on micturition, refusal to feed and weight loss. He looked ill and was in respiratory distress, irritable, febrile (38.8 °C), moderately dehydrated and pale. His weight and length were 5.5 kg and 64 cm. He had a tender, firm and ballotable abdominal mass on the left flank measuring 8 cm × 10 cm. His pulse rate was 140 beats/min, blood pressure 60/40 millimetres of mercury and respiratory rate was 65 cycles/min. He had widespread coarse crepitations and normal heart sounds on chest auscultation.

**Management and outcome:**

An initial diagnosis of sepsis was made. Other considerations were nephroblastoma and neuroblastoma. Ceftriaxone and blood transfusion were commenced with subsequent administration of intravenous fluids. Further radiologic investigations revealed emphysematous pyelonephritis. The patient had percutaneous drainage and extended spectrum β-lactamase-producing *Escherichia coli* (sensitive to meropenem) which was isolated from the aspirate culture after 48 h of incubation. Meropenem could not be commenced because of non-availability and high cost. The patient subsequently deteriorated and died from septic shock.

**Conclusion:**

Emphysematous pyelonephritis has a fulminant course when not diagnosed promptly and treated adequately.

## Introduction

Emphysematous pyelonephritis (EPN) is a severe necrotising and progressive infection of the kidneys which is characterised by the formation of gas within the renal parenchyma, collecting system, or the perinephric tissue.^1^ It is rare in children, with the majority of cases occurring among adults with diabetes mellitus.^2^ Gas-forming organisms are said to be the causative organisms of which *Escherichia coli* is the most common.^3^ The pathogenesis is unclear but factors thought to increase predisposition to developing EPN include decreased host immunity, increased levels of glucose in the tissue, impaired tissue perfusion, obstruction of the urinary system and presence of gas-forming organisms in the host tissue.^1^ The diagnosis of EPN requires clinical features supported by radiologic investigations and isolation of the offending organisms. Depending on the stage of the disease, treatment could be medical alone or a combination of medical and surgical interventions.^1^

## Ethical considerations

Ethical approval to conduct the study was obtained from UDUTH Health Research Ethics Committee with registration number NHREC/30/012/2019. Authors obtained permission from the caregivers to publish the clinical details of the patient.

## Case presentation

We report the case of Y.B (initials of infant used to retain anonymity), a 7-month-old male infant that was referred from a secondary health facility in Sokoto, Nigeria, to Usmanu Danfodiyo University Teaching Hospital (UDUTH), Sokoto in November 2019. He was brought by his parents to the Emergency Paediatric Unit of UDUTH on account of a 5-day history of fever and vomiting, and a 3-day history of progressively enlarging left-side abdominal mass which was noticed incidentally and said to be tender to touch. There was no preceding history of trauma and there were no masses on other body parts. There was associated excessive crying, crying on micturition, refusal to feed and weight loss. He was admitted at the referring hospital at the onset of illness where he had anti-malaria (artesunate), and antibiotic (cefuroxime) treatment, as well as a blood transfusion with no significant improvement, necessitating referral to UDUTH Sokoto 72 h later. Operating within a resource-constrained health system, UDUTH is the highest tertiary level healthcare facility within the state. Both of his parents have no formal education and are ‘petty traders’ with a combined average earning of 40 000.00 Nigerian naira ($132.00 United States dollars [USD]) per month.

Physical examination revealed an ill-looking child in respiratory distress, irritable, febrile (axillary temperature = 38.8 °C), moderately dehydrated, pale, anicteric acyanosed with no significant peripheral lymphadenopathy. His weight and length were 5.5 kg and 64 cm, while oxygen saturation (SPO_2_) was 89% in room air. He had a tender, firm and ballotable abdominal mass extending from the left lumbar region to the left iliac region, measuring 8 cm × 10 cm. The right kidney, liver and spleen were not palpable. He had normal male external genitalia and was not circumcised. His pulse rate was 140 beats/min, blood pressure 60/40 millimetres of mercury and respiratory rate was 65 cycles/min. Chest auscultation revealed vesicular breath sounds with widespread coarse crepitations and normal heart sounds. The neurologic examination was also normal. A clinical diagnosis of sepsis with focus on the chest and urinary tract with malaria was made. Other considerations were nephroblastoma and neuroblastoma.

## Management and outcome

**FIGURE 1:**
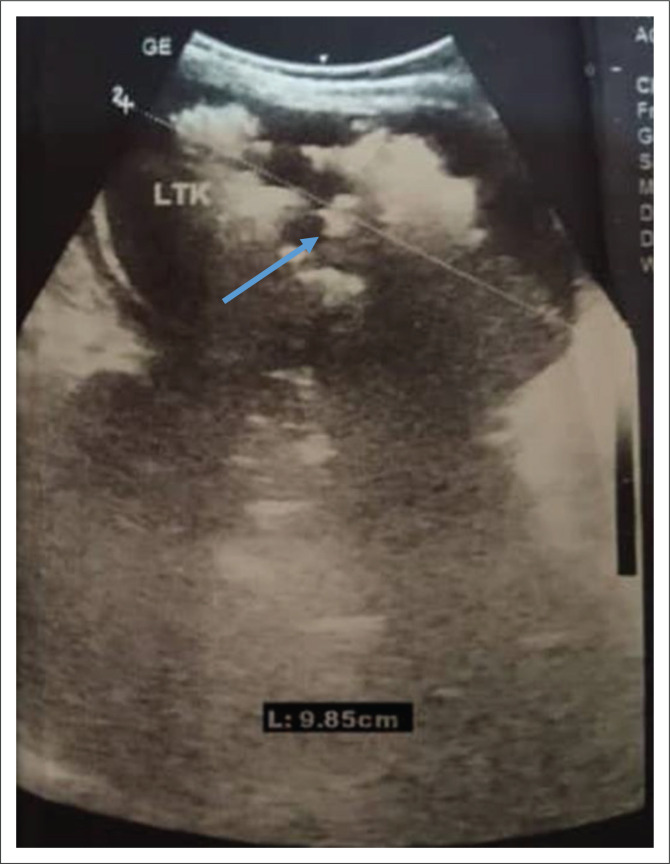
Sonogram of the left kidney of an emphysematous pyelonephritis patient at the Usmanu Danfodiyo University Teaching Hospital, Sokoto, Nigeria, November 2019. Figure shows renomegaly, reduced renal parenchyma echogenicity and multiple irregular mixed echoes with dirty shadowing (blue arrow).

**FIGURE 2:**
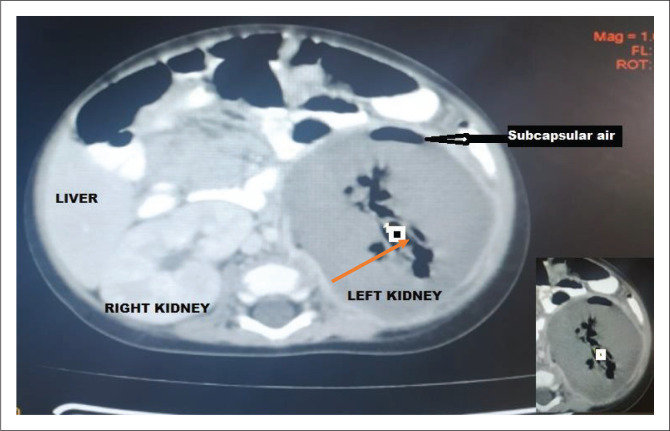
Contrast enhanced computed tomogram of an emphysematous pyelonephritis patient at the Usmanu Danfodiyo University Teaching Hospital, Sokoto, Nigeria, November 2019. Figure shows left renomegaly, contrast enhancement and multiple air densities within the calyceal system (orange arrow).

**FIGURE 3:**
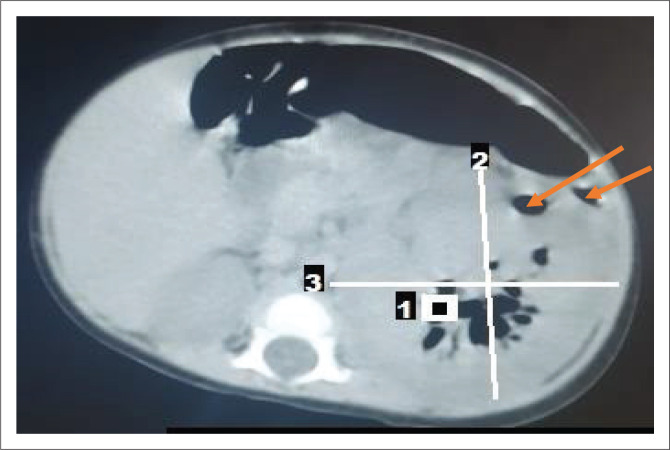
Non–contrast-enhanced computed tomogram of an emphysematous pyelonephritis patient at the Usmanu Danfodiyo University Teaching Hospital, Sokoto, Nigeria, November 2019. Figure shows left renomegaly with multiple air density area within the parenchyma and subcapsular region (orange arrows).

Broad spectrum empirical antibiotic (intravenous ceftriaxone) treatment was commenced empirically, and the patient was transfused with blood (haematocrit was 22%) and subsequently placed on intravenous fluids. Blood film for malaria parasite was negative, and metabolic panel and complete blood count were normal except for anaemia (Table 1). Urinalysis showed proteinuria, glycosuria and leucocyturia but urine microscopy and culture yielded no significant growth (Table 1). Human immunodeficiency virus DNA polymerase chain reaction was negative. Chest radiograph revealed multiple patchy perihilar opacities. Abdominal ultrasound demonstrated relative renomegaly of the left kidney with a bipolar length of 99 mm, turbid collection with multiple pockets of air within it and marked thinning of the parenchyma. The right kidney was normal in outline, position and size (72 mm in bipolar length) and other organs were normal in appearance (Figure 1). Computerised tomography (CT) scan of the abdomen revealed an enlarged left kidney with bipolar length and transverse diameters of 97 mm and 68 mm, reduced renal parenchyma enhancement (Hounsfield units = 36–42), while multiple oval and tubular negative density areas (Hounsfield units = 543–723) were noted centrally and in subcapsular regions suggestive of air in the collecting system and renal parenchyma with overall features in keeping with EPN. The right kidney was normal in position, outline and size (bipolar diameter 63.8 mm and transverse diameter 40.8 mm), no mass lesions or calculus were seen, and other organs were normal (Figure 2 and Figure 3). The patient’s diagnosis was changed to septicaemia complicated by unilateral (left) emphysematous pyelonephritis class II. Ceftriaxone treatment was continued while awaiting the culture result, and an ultrasound guided percutaneous drain was inserted, which drained about 60 mL of purulent, blood-stained material containing air bubbles. The result of aspirate microscopy, culture and sensitivity revealed numerous pus cells on microscopy, and growth of *E. coli* confirmed to be extended spectrum β-lactamase-positive via phenotypic method, which was sensitive only to meropenem. Antibiotic therapy was changed to intravenous meropenem but could not be commenced due to unavailability, as well as the high cost of the medication where available. Although there was reduction in his temperature to 37.9 °C, he subsequently developed shock and deteriorated rapidly. He did not respond to resuscitation and died 24 h later. Although not the recommended treatment for this case with stage II EPN, nephrectomy was considered but the rapidity in deterioration in the patient’s condition (shock and death within few hours) did not allow for adequate counselling and preparation for the procedure.

**TABLE 1:** Laboratory data of the emphysematous pyelonephritis patient on the second day of admission at the Usmanu Danfodiyo University Teaching Hospital, Sokoto, Nigeria, November 2019.

Metabolic panel	Blood count	Clotting profile	Urinalysis	Urine microscopy. culture, sensitivity
Sodium 136 mmol/L	White blood cells 11.5 × 10^9^/L	PT-test 16 s	Turbid	Pus cells +
Potassium 4.1 mmol/L	Lymphocites 7.2 × 10^9^/L	PT-control 14 s	Protein +	Yeasts
Chlorine 97 mmol/L	Granulocytes 3.5 × 10^9^/L	PTTK-test 32 s	Blood –	No growth
Bicarbonate 22 mmol/L	Haematocrit 22%	PTTK-control 31 s	Glucose +	-
Blood urea nitrogen 4.4 mmol/L	Platelets 123 × 10^9^/L	INR- 1.16	Leucocyte ++	-
Creatinine 0.5 mg/dL	-	-	Nitrite	-
Glucose 2.8 mmol/L	-	-	-	-

PT, prothrombin time; PTTK, partial thromboplastim time with kaolin; INR, international normalised ratio.

## Discussion

The 7-month-old male infant in this study had acute onset of fever, vomiting and a left-side ballotable abdominal mass with radiologic features of stage II EPN confirmed to be caused by extended spectrum β-lactamase *E. coli* on aspirate microscopy, culture and sensitivity. Emphysematous pyelonephritis is a severe necrotising infection of the renal parenchyma that causes gas accumulation within the renal tissues, with or without the involvement of the peri-renal spaces.^4,5^ Reports of EPN has been documented in adults, especially among diabetics, hypertensives and renal transplant recipients following end-stage renal disease.^6^ Women are said to be more at risk than men.^1,5^ Emphysematous pyelonephritis is rare among children with the first known paediatric case reported in a 10-year-old female in 1985.^7^ There are few reports of EPN among children in studies from South Africa,^8^ Texas^9^ and Saudi Arabia,^10^ with none of the patients having diabetes mellitus nor was there significant gender preponderance in the occurrence of EPN. However, one of the reported cases was a transplant recipient.^10^ The index patient is, to the best of our knowledge, the first documented case in a child in Sokoto State, and possibly in Nigeria. Unlike in previous reports^9,10^ in which cases had underlying medical conditions (chromosomal abnormality with ectopic right ureter, and end-stage renal disease from neurogenic bladder), our case was apparently healthy with no identifiable risk factors prior to the development of EPN. Possibly, our case is an indication that EPN can in very rare instances occur in previously apparently healthy children. The most common causative bacterial organism is *E. coli,* which is also the organism that was isolated from the aspirate specimen of our patient. Other pathogenic agents of EPN include *Klebsiella, Proteus, Pseudomonas, Citrobacter, Enterococcus* and *Streptococcus* species. Rare organisms such as coagulase negative *Staphylococcus, Clostridium, Candida* species and *Aspergillus fumigates* have also been reported.^1,3,9,10^ Unlike in our case, the study by Siddique et al.^9^ reported *Enterobacter cloacae* as the causative agents of EPN in their patient. The pathogenesis of EPN is not yet known; however, the formation of carbon dioxide from the fermentation of glucose in urine and kidney tissues is thought to be the main mechanism.^1^ The predisposing factors that have been implicated include the presence of a gas-forming bacterial organisms, high glucose levels in tissues, immunosuppression (e.g. diabetes and immunosuppressive therapy), urinary tract obstruction, and poor tissue perfusion.^1,11,12^ Local tissue ischaemia in the presence of a gas-producing pathogen is thought to exacerbate tissue destruction, encourage the production of pus, and inhibit the removal of locally produced gas, leading to EPN.^11^ Other speculated mechanisms are that the increased levels of glucose in the tissues together with decreased blood supply to the kidneys contributes to the anaerobic metabolism of glucose and lactate by the organisms and thereafter the production of gases like carbon dioxide, hydrogen, nitrogen, oxygen and methane by the gas-forming organisms.^13^ The clinical manifestation of EPN in children is similar to that of adults with the main features being fever, anorexia, nausea, vomiting, flank pain with or without palpable mass, and dysuria.^7,13^ Our patient presented with some of the aforementioned symptoms. The classification of EPN is via radiologic imaging, and the most commonly used classification system is by Huang and Seng using CT scan to classify EPN into five (1–4b) classes.^1^ Our patient’s CT scan findings (Figure 2 and Figure 3) placed him at EPN class II because the demonstrated air went beyond the left collecting system to involve the renal parenchyma. Although earlier studies^9,10^ reported renal ultrasonograms suggesting air collection in the renal parenchyma of their patients, EPN was not staged using a impossible CT scan in the studies making it impossible to compare the stage of EPN in our study with theirs. The clinical presentation, radiologic imaging and isolation of causative organisms confirms the diagnosis of EPN.^1^ Modalities of treating patients with EPN are said to have changed over time, with intensive conservative management assuming a prominent position, depending on the class of EPN and other co-morbidities. Broad spectrum antibiotic therapy with percutaneous drainage is the standard recommended treatment protocol for EPN classes I and II. The treatment options for patients with EPN classes III and IV depend on the presence and number of risk factors such as shock, acute kidney injury, thrombocytopaenia and coma. The choice of treatment for patients with fewer than two risk factors is antibiotics and percutaneous drainage. In the presence of two or more risk factors, nephrectomy is the recommended treatment.^1^ Our patient had percutaneous drainage but could not commence the appropriate antibiotic (meropenem) therapy due to non-availability and high cost. Although UDUTH is the highest tertiary level option within the state, meropenem is not indigenously produced to the best of our knowledge. Although available, his parents could not afford the supply of ten 500 mg powder for the injection, which cost 18 000.00 Nigerian naira ($59.00 USD).^14^

Unlike in our patient, other studies have reported good response to antibiotics like third-generation cephalosporin, fluoroquinolones and vancomycin.^6,8,9^ Good outcomes for EPN have been reported with prompt initiation of recommended treatment.^9^ However, EPN may run a fatal course if not identified and treated early.^4,5,9^ There was a delay in diagnosing the index case due to late presentation. Additionally, the isolated organism (extended spectrum β-lactamase *E. coli*) was resistant to the empirical antibiotic (ceftriaxone) therapy. The recommended antibiotic (meropenem) could not be commenced because of the aforementioned reasons. It is, therefore, not surprising that the patient succumbed to the illness.

### Conclusion

Our experience brings to the fore the occurrence of EPN in an infant and the fulminant course it can take when not diagnosed promptly and treated adequately. Our case highlights the importance of a high index of suspicion, the resistance of extended spectrum β-lactamase *E. coli* to the empirical antibiotic (ceftriaxone), and the need to ensure availability as well as cost effectiveness of recommended antibiotics in the study location. These measures will go a long way in the timely identification of cases and will improve the outcome of management of initial classes (1–3) of EPN.
